# Complement factor D regulates collagen type I expression and fibroblast migration to enhance human tendon repair and healing outcomes

**DOI:** 10.3389/fimmu.2023.1225957

**Published:** 2023-09-06

**Authors:** Junyu Chen, Jin Wang, David A. Hart, Zongke Zhou, Paul W. Ackermann, Aisha S. Ahmed

**Affiliations:** ^1^ Department of Orthopedics, Orthopedic Research Institute, West China Hospital of Sichuan University, Chengdu, Sichuan, China; ^2^ Department of Molecular Medicine and Surgery, Karolinska Institutet, Stockholm, Sweden; ^3^ The Key Laboratory of Tumor Molecular Diagnosis and Individualized Medicine of Zhejiang Province, Zhejiang Provincial People’s Hospital, Affiliated People’s Hospital, Hangzhou Medical College, Hangzhou, China; ^4^ Department of Pharmacology, School of Basic Medical Sciences, Xi’an Jiaotong University Health Science Center, Xi’an, Shaanxi, China; ^5^ McCaig Institute for Bone & Joint Health, University of Calgary, Calgary, AB, Canada; ^6^ Department of Trauma, Acute Surgery and Orthopaedics, Karolinska University Hospital, Stockholm, Sweden

**Keywords:** complement factor D, tendon rupture, dense connective tissue, collagen type I, proteomics, cell migration, patient outcome

## Abstract

**Introduction:**

Dense connective tissues (DCTs) such as tendon, ligament, and cartilage are important stabilizers and force transmitters in the musculoskeletal system. The healing processes after DCT injuries are highly variable, often leading to degenerative changes and poor clinical outcome. Biomarkers in relation to repair quality for human DCTs, especially tendon are lacking. This study expands our previous findings and aimed to characterize the mechanisms by which a potential biomarker of good outcomes, complement factor D (CFD), regulates tendon healing.

**Methods:**

Quantitative mass spectrometry (QMS) profiling of tissue biopsies from the inflammatory phase of healing (n = 40 patients) and microdialysates from the proliferative phase of healing (n = 28 patients) were used to identify specific biomarkers for tendon healing. Further bioinformatic and experimental investigations based on primary fibroblasts and fibroblast cell line were used to confirm the identified biomarkers.

**Results:**

The QMS profiling of tissue biopsies from the inflammatory phase of healing identified 769 unique proteins, and microdialysates from the proliferative phase of healing identified 1423 unique proteins in Achilles tendon rupture patients. QMS-profiling showed that CFD expression was higher during the inflammatory- and lower during the proliferative healing phase in the good outcome patients. Further bioinformatic and experimental explorations based on both inflammatory and proliferative fibroblast models demonstrated that CFD potentially improved repair by regulating cell migration and modulating collagen type I (Col1a1) expression. Moreover, it was shown that the enhanced Col1a1 expression, through increased fibroblast migration, was correlated with the validated clinical outcome.

**Discussion:**

The results of the current studies characterized underlying inflammatory- and proliferative healing mechanisms by which CFD potentially improved tendon repair. These findings may lead to improved individualized treatment options, as well the development of effective therapies to promote good long-term clinical outcomes after tendon and other DCT injuries.

**Trial registration:**

http://clinicaltrials.gov, identifiers NCT02318472, NCT01317160.

## Introduction

Dense connective tissues (DCTs) provide a matrix, which support and protect other tissues and organs in the human body, as well as acting as force transmitters in the musculoskeletal system. The healing process after an injury to DCTs such as tendon, ligament and meniscus, however, often leads to compromised healing with degenerative changes, pain and considerable individual variation in long-term clinical outcome. Comprehensive knowledge of the regulatory mechanisms responsible for the variation in outcomes in relation to specific biomarkers of DCT healing is still lacking (1–3).

In previous studies, evidence was presented for a central role for Complement factor D (CFD) in tendon healing and showed that CFD has prognostic efficacy for long-term clinical patient outcome after tendon injury (4). CFD, also known as adipsin and expressed by adipose cells (5), plays important role in both physiology and pathophysiology, with its’ regulatory function in the complement system (6). The complement system is activated as the first-line of defense against invading pathogens (7) but is also involved in systemic inflammation, thrombosis and even autoimmune diseases (8, 9).

Healing is a complex process including three overlapping stages, inflammation, proliferation, and remodeling (10), in which the complement system may have regulatory functions (4). Fibroblasts, the most common cells in many DCTs, produce a major building block of tendon collagen type I, alpha-1 (Col1a1), which in addition to bringing structure and strength to the matrix can act chemotactically to induce fibroblast migration (11, 12). Higher Col1a1 production is reported to be a key reason for improved DCT healing (13). However, whether CFD has a direct role in Col1 production or/and indirectly via effects on fibroblast migration to the wound site following a tendon injury remains mainly unknown.

In this study, we aimed to characterize the association between CFD and human tendon repair during both the inflammatory- and proliferative stages of healing by combining two quantitative mass spectrometry (QMS)-based proteomic profiles with a validated clinical outcome database. Additionally, this study aimed to investigate the relationship between CFD and Col1a1 expression during the inflammatory- and proliferative phases of healing based on available human tissue samples and further explored the detailed mechanisms using *in vitro* studies.

## Materials and methods

### Clinical samples and data collection

#### Patient eligibility criteria and randomization

The database included patients diagnosed with acute ATR and surgically repaired at the Karolinska University Hospital. A total of 55 patients were randomly selected from 2 previously conducted randomized control trials (RCT) and were dichotomized into two clinical cohorts according to their outcome based on Achilles Tendon Rupture Score (ATRS) (**Table 1**). All patients in this study exhibited mid-substance ATR that were surgically repaired within 2-7 days of injury. During surgery, the inflammatory phase biopsies were collected from a subgroup of patients (n=40) and at two weeks post-surgery during the proliferative healing phase, injury site microdialysates were collected from a subgroup of patients (n=28). Both biopsies and microdialysates were collected from 13 patients, therefore the total number of 55 patients.

**Table 1:** Basic information and characteristics of all the participants.

	Group	Good Outcome	Poor Outcome	p-value
Microdialysate	28	n=14	n=14	
Sex (M:F)	21:7	11:3	10:4	ns
Age (years)	37 (32-42)	36 (29-39)	42 (33-48)	0.049
BMI (kg/m2)	25.6 (24.1-28.1)	25.5 (26.7-24.2)	26.3 (28.1-23.7)	ns
ATRS	78 (62-92)	94 (91-100)	58 (50-73)	<0.0001
Limitation in calf strength	8 (5-10)	9 (8-10)	5 (3-7)	<0.0001
Tiredness in the calf	9 (7-10)	10 (9-10)	7 (5-8)	<0.0001
Stiffness in the calf	9 (6-10)	10 (9-10)	7 (5-9)	0.001
Pain in the calf	9 (7-10)	10 (10-10)	7 (4-9)	<0.0001
Activity of daily life (ADL)	9 (7-10)	10 (10-10)	7 (5-9)	<0.0001
Walking on uneven surface	9 (7-10)	10 (9-10)	7 (5-9)	0.003
Limitation on walking in stairs	9 (8-10)	10 (9-10)	8 (5-9)	0.001
Limitation on running	7 (4-10)	10 (9-10)	5 (3-7)	<0.0001
Limitation on jumping	9 (5-10)	9 (8-10)	5 (3-6)	<0.0001
Loss in physical work	8 (8-10)	10 (10-10)	8 (5-8)	<0.0001
HRT (%)				
LSI Time and power	82 (73-95)	89 (79-97)	78 (66-83)	ns
LSI Total work	71 (60-89)	85 (69-102)	70 (55-78)	ns
LSI Repetition	92 (73-101)	100 (96-104)	81 (68-94)	0.018
LSI Average height	80 (13-88)	88 (79-98)	78 (68-83)	ns
Tendon biopsies	40	n=20	n=20	
Sex (M:F)	32:8	20 (17:3)	20 (19:1)	ns
Age (years)	40 (26-65)	40 (26-65)	39 (28-59)	ns
BMI (kg/m2)	25.4 (19.6-34.7)	24.6 (19.6-34.7)	25.4 (20.6-30.6)	ns
TTS (h)	85.8 (24.5-158.7)	87.5 (24.5-158.7)	85.3 (41.0-135.6)	ns
ATRS	76 (40-100)	95 (87-100)	63 (40-78)	<0.001
Limitation in calf strength	8 (1-10)	9 (7-10)	5 (1-8)	<0.001
Tiredness in the calf	8 (3-10)	10 (7-10)	5 (3-8)	<0.001
Stiffness in the calf	8 (2-10)	10 (5-10)	5 (2-8)	<0.001
Pain in the calf	10 (3-10)	10 (9-10)	9 (3-10)	<0.001
Activity of daily life	9 (4-10)	10 (9-10)	8 (4-10)	<0.001
Walking on uneven surface	10 (3-10)	10 (7-10)	8 (3-10)	<0.001
Limitation on walking in stairs	9 (4-10)	10 (9-10)	8 (4-10)	<0.001
Limitation on running	8 (0-10)	10 (8-10)	6 (0-9)	<0.001
Limitation on jumping	7 (2-10)	10 (7-10)	4 (2-9)	<0.001
loss in physical work	9 (3-10)	10 (9-10)	8 (3-10)	<0.001
HRT (%)				
LSI Time and Power	80.8 (23.5-189.9)	80.6 (52.9-115.9)	82.1 (23.5-189.9)	ns
LSI Total work	71.5 (24.3-288.0)	77.7 (57.5-119.6)	68.2 (24.3-288.0)	ns
LSI Repetition	90.5 (45.8-275)	96.7 (71.4-114.3)	82.7 (45.8-275.0)	ns
LSI Average height	81.8 (37.6-110.5)	81.8 (65.1-110.5)	81.9 (37.6-104.0)	ns

The clinical and functional characteristics of ATR patients included in the study. BMI, body mass index; ATRS, Achilles tendon Total Rupture Score (0-100, worst = 0); HRT, Heal Rise Test (0-100, worst = 0 for each category); QOL, Quality of Life; LSI, Limb Symmetry Index). Data presented as median with lower and upper interquartile ranges. Comparison between good versus poor outcome was measured by Mann-Whitney U test. A p value < 0.05 was set for statistical significance between group. n, no significance.

Exclusion criteria used in the RCTs included inability to provide verbal and/or written consent for participating in the study, current anticoagulation treatment, allergic to contrast liquid, known renal failure, heart failure with pitting oedema, thrombophlebitis or known coagulation disorder, inability to follow instructions or were planned for follow up at other hospitals, received other surgery during the month before tendon rupture, and patients with known malignancy, haemophillia or pregnancy.

#### Surgery and biopsy collection

The same anesthetic and surgical techniques were used for all patients using a predefined study protocol, as described earlier (14). Local anesthetic was administered (20 ml of Marcain^®^ and adrenalin 5 mg/ml, AstraZeneca, London, UK) in the dermis, subcutis and peritendinous space prior to surgery. The patients were subsequently placed in the prone position and a medial incision was made through the skin, fascia cruris and paratenon. From the ruptured area, the surgeon retrieved Achilles tendon biopsies. The tendon ends were sutured together using a modified Kessler suture with two 1–0 polydioxanone (Ethicon, Somerville, New Jersey, USA) sutures. The paratenon and fascia cruris were then closed with 3–0 Vicryl (Ethicon, Somerville, New Jersey, USA) and the skin was sutured with 3–0 Ethilon (Ethicon, Somerville, New Jersey, USA).

#### Microdialysate collection

Microdialysates were collected at 2-week post-surgery from the operated and non-operated healthy leg as described previously (15). Briefly, a microdialysis catheter (CMA Microdialysis AB, Sweden) with a 100 kDa molecular cut-off, was introduced into the peritendinous space 2-5 mm ventral to the Achilles tendon. A perfusion fluid (Macrodex) was pumped through the catheter at 1.0 μL/min speed and collected in vials every 30 min for 2 hours. Owing to the lingering effect of the insertion trauma and the possible differences due to pump adjustment, the first of the four vials was not considered reliable and therefore was not included in the analysis. Earlier studies have verified that this methodology can assess increased metabolism, as well as matrix deposition (16) in the healing compared to the healthy contralateral Achilles tendon. Prior studies have moreover demonstrated that peritendinous microdialysates from the proliferative healing phase in patients with ATR can capture specific tendon substances, which are predictive of patient outcome at one year (16, 17). Thus, the microdialysate technique was used to identify protein expression during the proliferative healing phase.

#### Patient-reported outcome

Patient-reported outcomes were assessed by the validated questionnaire, Achilles Tendon Total Rupture Score (ATRS), at one-year post-surgery during the follow up. The ATRS consists of 10 specific sub-scales which includes strength of tendon, tiredness in the tendon, stiffness in tendon, pain in tendon, as well as limitations in activity of daily life (ADL), limitation on uneven surface, in stairs, when running, when jumping and loss in physical work. Each sub-scale ranges from 0 to 10 where 0 = worst and 10 = best outcome with no limitation. The maximum total score of ATRS is 100 and a score higher than 80 was regarded as good subjective outcome (18).

#### Functional outcome

At 1-year post-surgery, functional outcomes of all patients were evaluated by using the heel-rise test (HRT), a validated method which has been used previously (16, 19, 20).

The HRT was performed on one leg at time with patient standing on a box with 10° incline wearing standardized footwear connected to a linear encounter, with the test sequence being 30 per minute using a metronome. Patients were told to perform as high, and as many heel-rises as possible. The test was stopped when the patient could no longer perform a complete heel-rise or maintain the sequence of 30/min. All results, including the number of heel-rises, height of every single heel-rise, total work in joules (total distance × body weight), time and the power (work/time) were recorded for further data analysis. The Limb Symmetry Index (LSI) was calculated to show the ratio between injured and contralateral healthy leg and is presented as a percentage (injured/contralateral healthy X 100).

### Quantitative mass spectrometry

#### Protein extraction from tissue biopsies and microdialysate

Tissue samples were solubilized in urea and NaCl with ProteaseMAX (Promega) in ammonium bicarbonate (AmBic) and mixed vigorously. Furthermore, samples were quickly frozen on block and subjected to disruption with a Vortex Genie disruptor before additional NaCl, urea and AmBic were mixed in. The supernatant fraction was transferred to new tubes after centrifugation at 10000 x g and at 4°C, AmBic was again added, and the tubes were vortexed vigorously for 2 minutes.

For assessment of microdialysates, the proteins were reduced with dithiothreitol in AmBic and then alkylated with iodoacetamide in AmBic in the absence of light. Trypsin (sequencing grade, Promega) was used to prepare proteolytic digestions of microdialysate proteins overnight. After adding concentrated formic acid, the reaction was stopped. Samples were subsequently cleaned and dried using a vacuum concentrator (miVac, Thermo scientific).

#### Reversed-phase liquid chromatography-mass spectrometry/MS analysis

Briefly, a C18 EASY-spray and C18 trap columns linked to an Ultimate 3000 UPLC system (Thermo Scientific) were used for the reversed phase liquid chromatographic separations of peptides. An Q Exactive HF mass spectrometer (Thermo Scientific) was used for the subsequent mass spectra, followed by data-dependent higher-energy collisional dissociation (HCD) fragmentations from precursor ions with a charge state.

#### Protein identification and quantification

The raw data were analyzed using the Mascot v2.5.1 (MatrixScience Ltd., UK) search engine. The Human Uniport database (last modified: 3 September 2020; ID: UP000005640; 75,777 proteins) was matched with the MS/MS spectra using the MSFragger database engine (21).

#### Cell culture

In order to evaluate the healing mechanisms of CFD in dense connective tissue repair fibroblast cells were used. The use of fibroblast cells for tendon research has been previously reported (22). For *in-vitro* studies, human primary fibroblasts (Promo Cells) and the fHDF/TERT166 fibroblast cell line (Evercyte, Austria) were used to investigate the effect of CFD on Col1 expression. Cells were cultured in Dulbecco’s Modified Eagle Medium/F12 (DMEM/F12, Gibco) supplemented with 10% fetal bovine serum (FBS, Gibco) and 1% penicillin-streptomycin (PEST, Gibco) at 37°C in an incubator with a humidified atmosphere containing 5% CO_2_. Cells were dissociated with 0.25% trypsin- ethylenediaminetetraacetic acid (Trypsin-EDTA) (ThermoFisher Scientific) and seeded in 12-well plates with 2.5 x 10^5^ cells/well.

#### Inflammatory fibroblast model creation

Recombinant human tumor necrosis factor (TNF) (Pepro Tech) was used to establish an inflammation-induced injury model after transfection of cells. Model creation used TNF in 0.2% BSA at a concentration of 10 ng TNF/ml (23).

#### Cell transfection

Cells were seed in 12-well plates and incubated with CFD silencing RNA (siRNA CFD) to detect whether CFD impacts Col1a1 production. Fibroblasts were incubated with a final concentration of 100 nM siRNA CFD mixed with Lipofectamine™ 3000 transfection reagent (Thermo Fisher Scientific), Opti-MEM reagent was used for dilution.

#### Western blot analysis

Briefly, 5μg protein lysed from fibroblasts were loaded into each well of the gel. Proteins were separated using 4-12% Bis-Tris gel electrophoresis (Invitrogen), and then transferred to nitrocellulose membranes. Nonspecific binding was blocked by incubating the membranes using 5% nonfat milk diluted in 1x tris-buffered saline with 0.1% tween (TBST). Next, the membranes were treated with anti-CFD and anti-Col1a1 overnight at 4°C and then with a secondary antibody conjugated to HRP at room temperature. The chemiluminescence signal was identified and quantified by Image Lab (Bio-Rad ChemiDoc MP Imaging System) where the immune-positive bands were normalized with the band for the house-keeping gene, beta-actin (β-actin).

#### Statistical analysis

SPSS (IBM SPSS, v26.0), GraphPad Prism 8.0 and R were used for statistical analysis and data plotting. All the variables were checked for skewness. Standard descriptive statistics were used to summarize clinical variables as means and standard deviations. Mann-Whitney *U* test was used to calculate differential expression of proteins among subgroups (good and poor outcome as well as healing and healthy), a *p* value < 0.05 and FC > 2 were considered as statistical significance. Univariate analysis was performed to check the association between a patients’ clinical outcome and the proteomic profiles. Outcome measurements which were significant in univariate analysis were subsequently used as the dependent variable in a multiple-linear regression model with independent variables, and a *p* value < 0.05 was set as significant threshold.

## Results

### Patient cohort

To establish a tissue atlas relating to good and poor patient outcome, tendon biopsies were collected from 40 ATR patients at the time of surgery during the inflammatory healing phase which represented 2-7 days after injury. Among these patients, 20 patients were assessed with good clinical outcomes and the other 20 with poor outcomes according to their 1-year validated patient-reported outcomes (18). To assess the healing response during the proliferative healing phase, microdialysates were collected at 2 weeks after surgery from 28 ATR patients, including 14 with good and 14 with poor clinical outcomes. In 13 patients, both biopsies and microdialysates were retrieved and therefore the total number of patients was 55. Patient characteristics and their 1-year clinical outcome are presented in **Table 1**.

### Bioinformatic and prognostic detection of complement factor D during tendon repair

**Figure 1:**
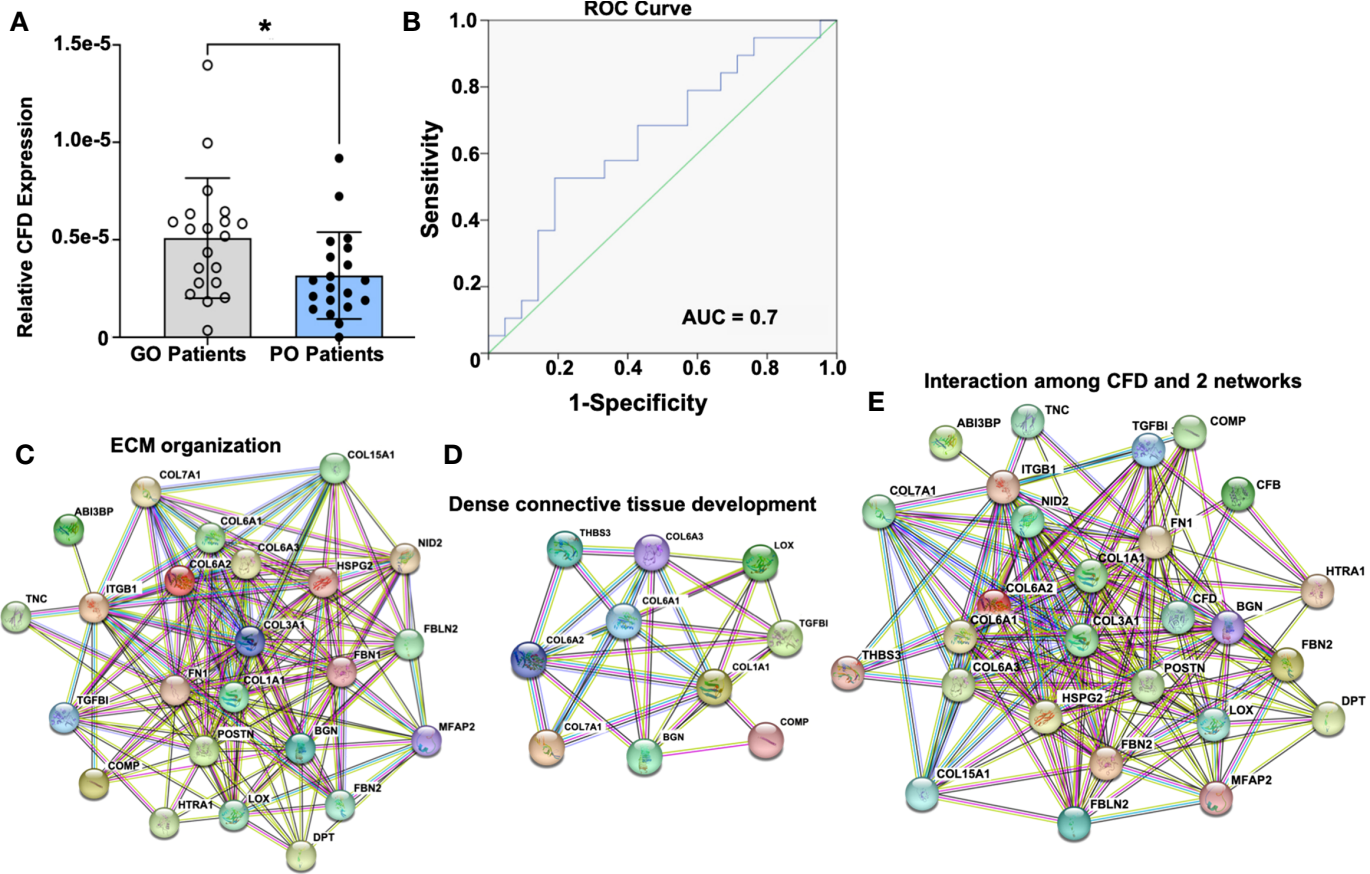
Prognostic significance of CFD and its potential role in tissue regeneration based on the inflammation-related proteomic file. **(A)** CFD expression in good outcome (GO) and poor outcome (PO) patients, n=20 in each group, Comparison between groups was measured by Mann-Whitney *U* test. A *p* value < 0.05 was set for statistical significance between groups.; **(B)** Predictive detection of CFD by using AUC model; CFD interaction with proteins involved in **(C)** extracellular matrix organization; **(D)** dense connective tissue development; **(E)** combined with all proteins related to tissue regeneration based on GO annotation and KEGG database. *p < 0.05.

In total, 769 unique proteins were identified from the biopsies as representative of the inflammatory stage of healing, whereas 1423 proteins were detected from microdialysates representing the proliferative healing phase of DCT repair. After combining proteomic profiles from both phases, CFD was detected to be significantly associated with patient outcome in both the inflammatory and proliferative healing stages, although with opposing effects. Interestingly, elevated CFD levels were detected during the inflammatory phase (**Figure 1A**) while decreased expression was observed during the proliferative healing phase (4) in good compared to poor outcome patients. To identify the prognostic significance of CFD during DCT repair, we combined the proteomic profiles together with the validated clinical database utilizing a multiple-regression model. As a result, CFD was detected to be significantly associated with patient outcome in both the inflammatory and proliferative healing stages, although with opposing effects.

At the inflammatory healing stage, higher CFD expression was observed in good outcome patients (**Figure 1A**). To examine the prognostic significance for CFD an area under the curve (AUC) model was established. An AUC value of 0.7 was identified for the inflammation stage (**Figure 1B**) indicating high-quality significance of CFD as a prognostic biomarker of healing. These observations were in accordance with our previous findings where CFD, during the proliferative stage of healing, was identified as a prognostic biomarker, however, with decreased expression among good outcome patients (4). Thus, significant opposite levels of CFD were observed in good outcome patients when compared with poor outcome patients is dependent on the healing stage, i.e. inflammatory and proliferative healing.

In the next step, all the differentially expressed proteins considered as healing-related biomarkers between good and poor outcome patients at both phases were used for the detection of biological processes or pathways based on GO annotation and KEGG database. At the inflammation stage, two highly enriched protein-protein interaction networks were detected, which were associated with extracellular matrix (ECM) organization (**Figure 1C**) and DCT development (**Figure 1D**). Interestingly, CFD was found to have strong interactions with both of these networks, demonstrating its potential central role during ATR repair (**Figure 1E**). Similar protein-protein interactions were identified for CFD during the proliferative healing phase as reported previously by us (4). CFD´s interaction in ECM organization together with its high reliability and sensitivity for predictive efficiency at both the inflammatory and proliferative healing stages, indicated a significant role for this biomarker during ATR healing.

### Association between CFD and healing related biomarker Col1a1 in patient samples

**Figure 2:**
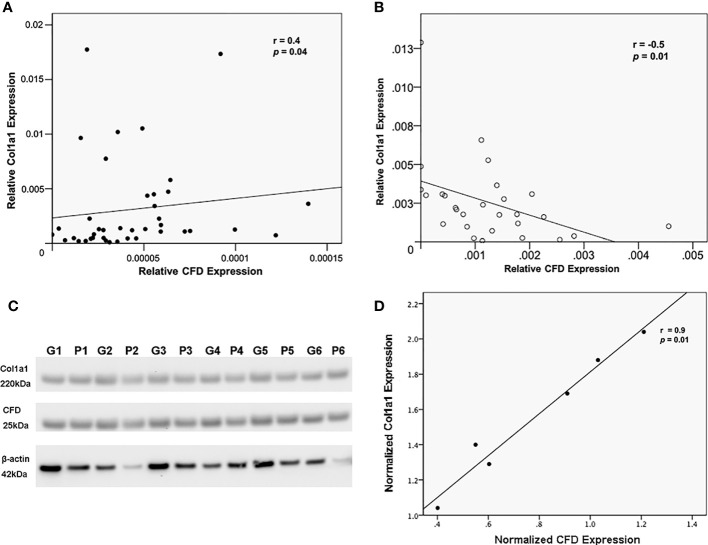
The relationship between CFD and Col1a1. Association of CFD and Col1a1 expression as measured by MS in, **(A)** tissue biopsies as representative of the inflammatory healing stage, and **(B)** microdialysate as representative of proliferation healing stage; **(C)** Western blotting analysis of CFD, Col1a1 and beta-actin (β-actin, a house keeping gene) expression in tissues collected from good (G) and poor (P) outcome patients, n = 6 in each group; **(D)** confirmative association analysis between CFD and Col1a1 in biopsies by using their normalized expression level in Western blot.

Our stepwise regression analysis identified a correlation between CFD with other healing-related markers, especially with different collagens. This is of interest as collagens are some of the most vital cellular components for maintaining and remodeling of the AT matrix. Interestingly, we observed opposite associations among CFD and collagen type I. Thus, CFD was positively associated with Col1a1 at the inflammation phase (**Figure 2A**), while negatively correlated to Col1a1 at the proliferative stage of healing (**Figure 2B**). Confirmative experiments based on Western blot analysis (**Figures 2C, D**) also supported the proteomic data findings acquired during the inflammation phase, and likewise the meso-scale detection at the proliferation stage as reported previously (4).

### Association between CFD and Col1a1 expression in inflammatory and proliferative *in vitro* models

**Figure 3:**
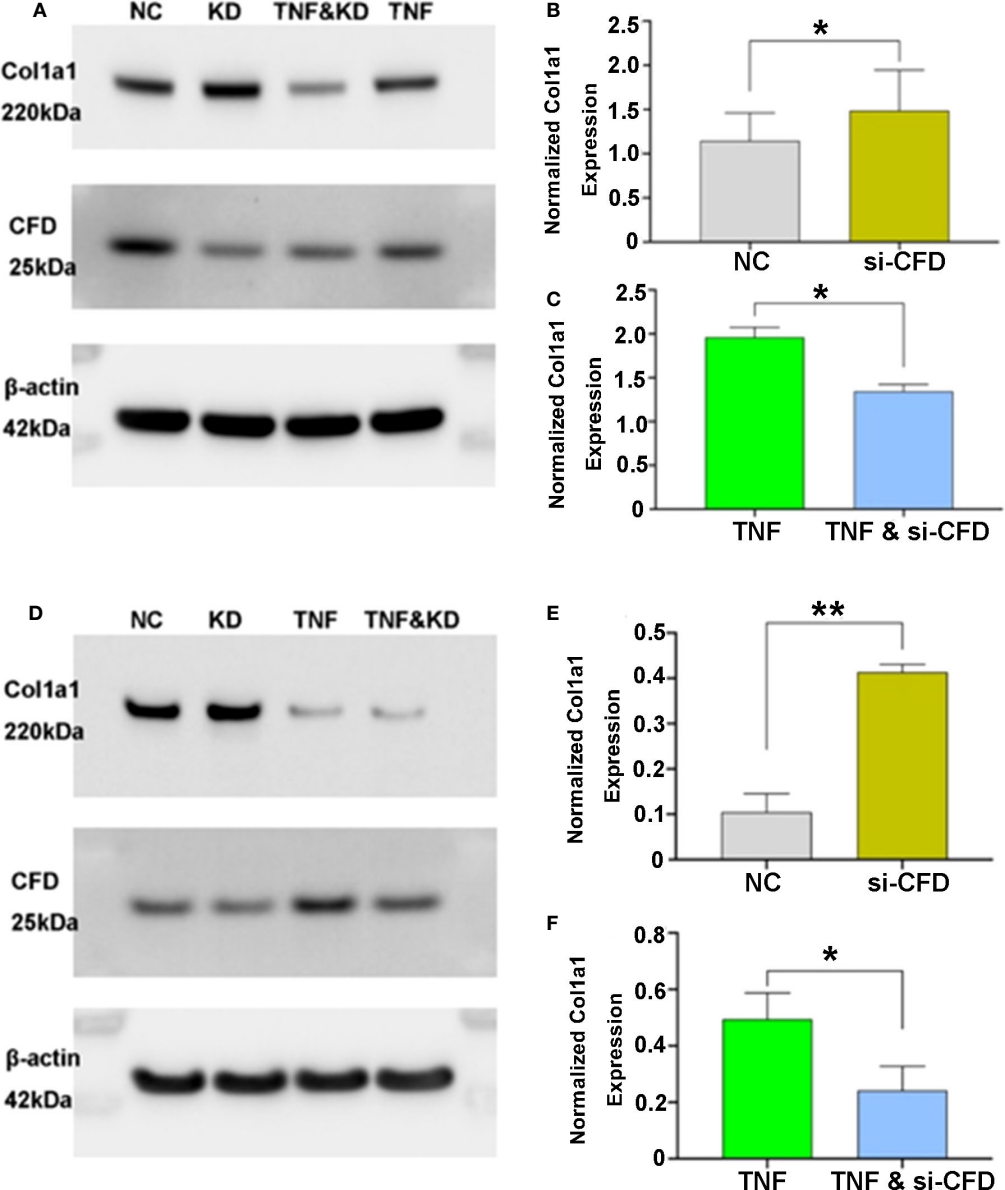
CFD regulation of Col1a1 expression in *in vitro* models of the inflammatory and proliferative phases of healing. Knock-down (KD), silencing (si) of CFD regulates Col1a1 expression during inflammation (TNF-induced) and proliferation (NC, normal challenge) among **(A–C)** primary human fibroblasts and **(D–F)** a human fibroblast cell line. Signal intensity was used for semi-quantitative analysis of western blot obtained proteins, and the intensity of the house-keeping gene (beta-actin) used for normalization. Data were reported with mean ± SD, *p < 0.05, **p < 0.01, 3 replicates were used for quantitative analysis.

After tendon injury, inflammation is the first phase of tissue repair, subsequently followed by the proliferation phase. To confirm and investigate the effect of CFD on Col1a1 expression during healing, inflammation-induced and unchallenged fibroblast injury models based on human primary fibroblasts and a fibroblast cell line were assessed. After gene silencing treatment, Col1a1 production was significantly lower when CFD was knocked down in the inflammatory fibroblast model. In contrast, knockdown of CFD in the unchallenged model led to an increased Col1a1 synthesis, indicating that the lower expression of CFD during the proliferation stage potentially improves tendon healing by up-regulation of Col1a1 production. The findings were confirmed both in the primary fibroblasts (**Figures 3A–C**) and in the fibroblast cell line (**Figures 3D–F**), strengthening the concept that CFD can affect Col1a1 synthesis during the healing response.

### CFD Enhances tendon repair by improving human fibroblast migration

**Figure 4:**
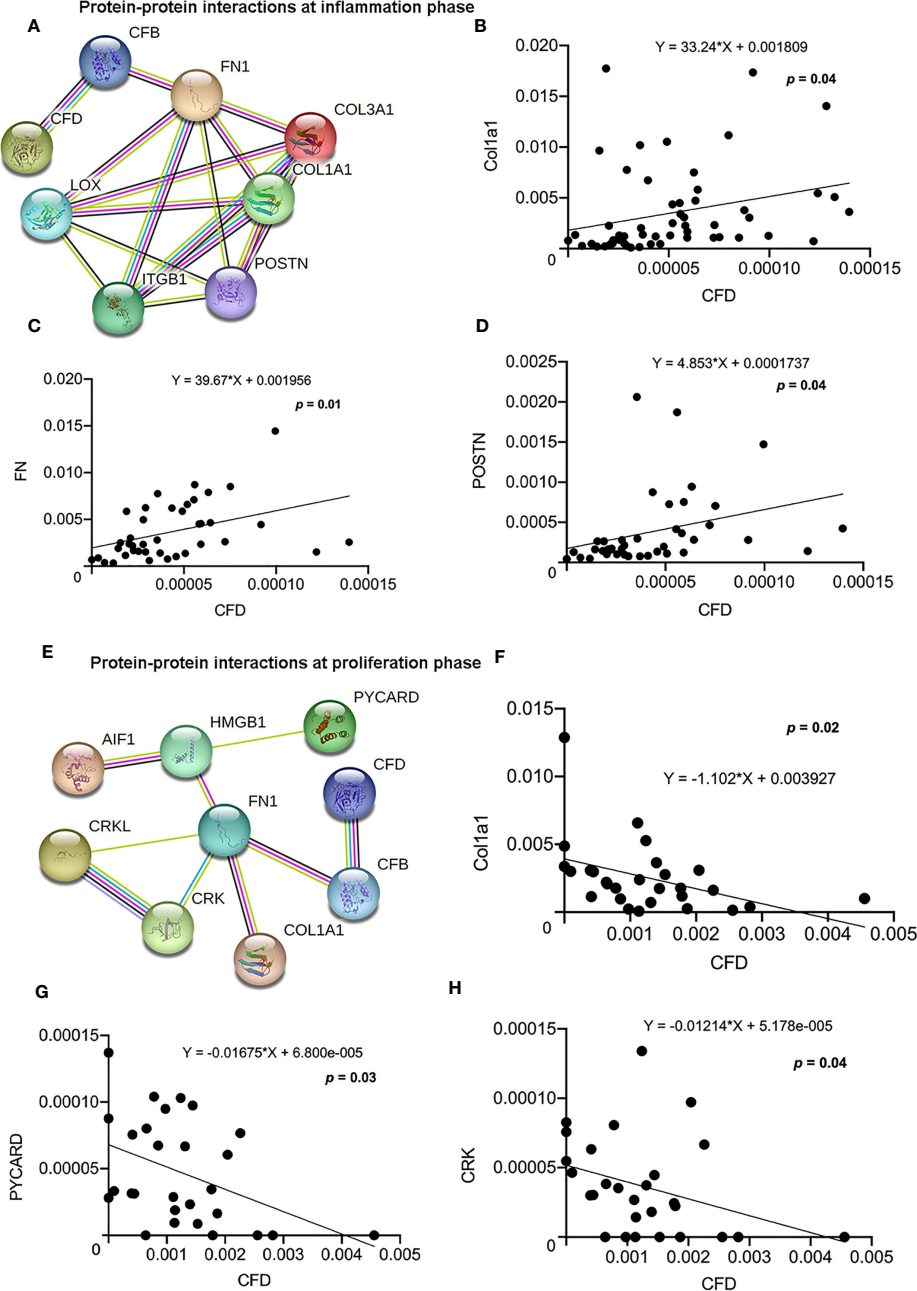
Protein-protein interactions and association among CFD and highly enriched migration-related proteins. **(A)** Migration network at the inflammation phase. The association between CFD and **(B)** Col1a1; **(C)** FN and **(D)** POSTN as analyzed by regression analysis. **(E)** Migration network at the proliferation phase. The association between CFD and **(F)** Col1a1; **(G)** PYCARD and **(H)** CRK analyzed by regression analysis, *p* < 0.05 indicates statistical significance.

The migration of fibroblasts to the site of injury is vital for the initiation of wound healing processes (24). Our GSEA analysis of the proteomic data sets from tissue biopsies representing inflammation and the microdialysates as representative of proliferation healing phase, identified cell migration as the most significant biological process among all the CFD related pathways. Further, we observed two highly enriched protein-protein interaction networks among migration associated proteins including both CFD and Col1a1, with many identical proteins, during the inflammatory (**Figure 4A**) and proliferative (**Figure 4E**) healing phases, respectively. The regression analysis further confirmed these interactions and identified significant associations for CFD with Col1a1, fibronectin 1 (FN1) and periostin (POSTN) at the inflammation phase (**Figures 4B–D**) and for CFD and Col1a1, apoptosis-associated speck-like protein containing a CARD (PYCARD) and CRK proto-oncogene adaptor protein (CRK) at the proliferation healing phase (**Figures 4F–H**).

**Figure 5:**
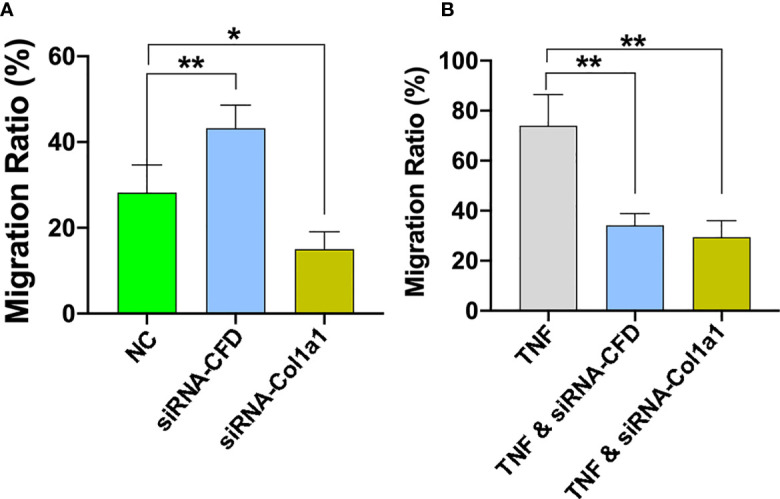
CFD regulates fibroblast migration during *in vitro* conditions that mimic both the inflammation and proliferation phases of healing. Silencing (si) of CFD regulates the cell migration rate following TNF-induced activation (inflammation) and in the absence of TNF (NC, un-challenge). Representative images and semi-quantitative analysis of cell migration rate assessed at 0 and 24 hours in **(A)** TNF-induced inflammation phase and **(B)** un-challenged, with, without siRNA CFD and treated with siRNA-Col1a1. Data reported as mean SD, *p < 0.05, **p < 0.01, n =3 replicates.

To confirm the role of CFD on fibroblast migration, a gene silencing technique in *in vitro* wound models was used. For these experiments, a scratch was created in inflammatory and unchallenged monolayer cultures of the primary fibroblasts and in the fibroblast cell line treated with or without siRNA CFD. The findings demonstrated that knock down of CFD decreased the fibroblast migration ratio (27%) following TNF activation, while the migration ratio was increased (52%) in the absence of TNF in primary fibroblasts and in the fibroblast cell line (**Figures 5A, B**). Collectively, GSEA and regression analyses of proteomic data together with the *in vitro* experiments indicate that increased CFD levels, observed in good outcome patients during inflammation, enhances fibroblast migration regulated via a network of migration associated proteins, including Col1a1. Moreover, decreased CFD levels, observed in good outcome patients during the proliferation phase, also enhances fibroblast migration, in part by a similar network of migration associated proteins, still including Col1a1.

### Function of Col1a1 in regulating healing outcome

To analyze whether the effect of CFD on fibroblast migration act through a pathway involving Col1a1 further experiments were performed. Previous studies have indicated that Col1a1 and its proteolytic-derived peptides at sites of tissue injury may act as chemotactic factors to attract fibroblast migration and initiate repair of damaged tissue (12, 25, 26). Thus, from the bioinformatic analysis of the QMS output it was found that Col1a1 also acts as a migration-related protein, which presented a strong association- with and was highly affected by CFD. Further experiments based on TNF-induced and un-challenged fibroblast models reported that the cell migration was reduced by knocking down of Col1a1 (**Figures 5A, B**). The findings presented indicate that CFD can regulate fibroblast migration, potentially via chemotactic control of Col1a1 that is differentially regulated by CFD depending on healing stage, to subsequently improve ATR repair and leading to enhanced patient outcomes 1-year after injury.

**Figure 6:**
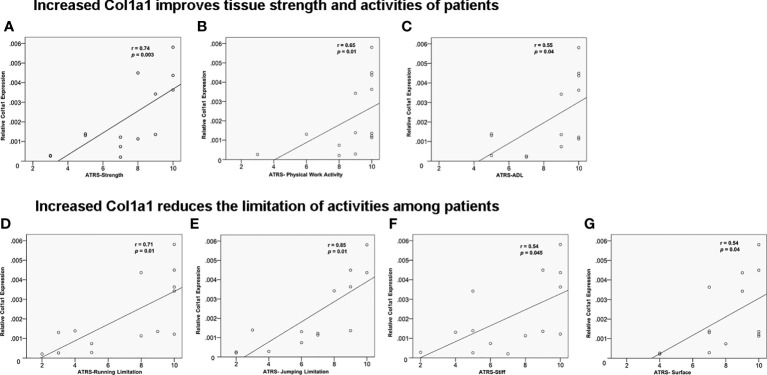
The effect of Col1a1 on improving 1-year clinical outcomes. Higher Col1a1 expression improved **(A)** tendon strength; **(B)** physical work activity and **(C)** activity of daily life. ATR subscale (0-10, worst = 0). Higher Col1a1 production related to CFD-induced fibroblast migration reduces the limitation of- **(D)** running; **(E)** jumping; **(F)** stiffness; **(G)** walking on uneven surface.

**Figure 7:**
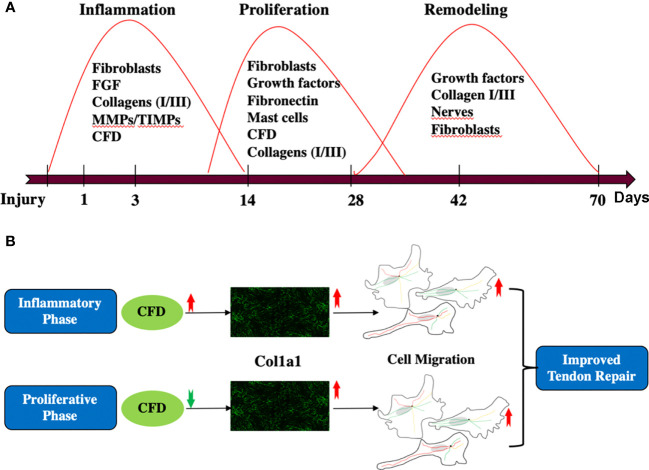
**(A)** A general review of the whole tendon healing process; **(B)** the role of CFD and Col1a1 during inflammatory and proliferative phase of healing.

To further support the clinically relevant role of elevated Col1a1 levels at the inflammatory healing stage, the effect of Col1a1 in the proteomic data set from tissue biopsies on clinical healing outcomes was further explored. The results strengthened a potential central function of CFD in tendon repair via inflammation-increased Col1a1 expression by demonstrating that a higher Col1a1 tissue levels improved tendon strength (**Figure 6A**), enhanced physical work activities (**Figure 6B**) and ADL (**Figure 6C**). Moreover, higher Col1a1 expression also reduced patients’ limitations in running (**Figure 6D**), jumping (**Figure 6E**), stiffness (**Figure 6F**) and walking on an uneven surface (**Figure 6G**). A summary of the potential roles of CFD and Col1a1 during the inflammatory and proliferative healing phases were reported in **Figure 7**.

## Discussion

In this study, the proteomic composition of good versus poor ATR repair was quantitatively characterized during both the inflammatory and proliferative phases of healing in patients with acute ATR. By combining data from tissue biopsies representing the inflammatory healing stage from the first week after injury and microdialysate samples representing the proliferative healing phase obtained at 2 weeks after repair surgery, our previous and present findings identified CFD as a novel biomarker with strong predictive association with long-term clinical outcomes and potential role in improving tendon repair. Further bioinformatic analysis and mechanistic exploration demonstrated that CFD has a potential role in enhancing tissue healing by regulating Col1a1 synthesis to enhance fibroblast migration during *in vitro* conditions that mimic both the inflammatory and proliferative healing stages.

DCT repair, using ATR repair as an example, is a complex process that exhibits partly overlapping healing stages, including inflammation, proliferation and remodeling (10). To elucidate insights into healing mechanisms and the related biomarkers, a stepwise, temporal procedure to capture the proteomic expression from the inflammatory to the proliferative healing stages was followed. Previous findings by us have indicated that a lower expression of CFD during the proliferative healing stage was associated with improved clinical outcomes (4). The present findings provide further understanding of the temporal healing mechanisms of CFD, demonstrating that higher CFD levels positively stimulated Col1a1 expression during the early inflammation phase and that lower CFD increased Col1a1 synthesis during the proliferative healing stage. Col1a1 is the major element of collagen type I, which is the most abundant protein in the ECM of most connective tissues, including tendons (27, 28). Previous findings have demonstrated that higher levels and faster production of Col1a1 is a key element of improved healing of such tissues (13).

Thus, one of the main findings presented is that higher CFD levels during the inflammatory phase induce increased Col1a1 expression, which leads to an enhanced fibroblast migration, and thereby promotes long-term healing quality. Previously, it has been shown that FGF also can serve as a prognostic biomarker during the inflammatory phase (3), and that FGF also can enhance Col1 expression (29). Therefore, there may be multiple factors that can regulate Col1 expression during different phases of healing. The tissue biopsies in this study were taken 2-7 days after injury, which is representative of the inflammatory phase of healing (30). The *in vitro* experiments additionally confirmed that the inflammatory stimulation induced by TNF presents a reduced Col1a1 production. However, the decreased Col1a1 synthesis was reversed by CFD. Based on these combined findings, we conclude that increased CFD levels during the inflammatory healing phase, at least until 1 week after injury, stimulates Col1a1 expression in the initial phase of tendon repair.

Although the exact pathways by which CFD regulates healing are not known, it has been reported that CFD, a zymogen, exists in two isoforms with potentially different functions (5, 31). It was reported that MASP-1 generates 3 different mRNAs (MASP-1, MASP-3 and Map44) due to alternative splicing, and MASP-3 functionally cleave inactive Pro-factor D (ProFD) into active CFD (32). CFD subsequently participate in the alternative complement pathway which produces complement factor 3 and 5 (C3 and C5). Earlier studies have demonstrated that C3 and C5 improve wound healing by increasing vascular permeability, increased inflammatory cell recruitment, and subsequent fibroblast migration (33, 34). Thus, it might be downstream factors in the complement cascade, eg. C3 and C5, which are also potentially involved in the regulation of Col1a1 expression.

Whether the increased Col1a1 production, due to the regulation by CFD is the sole regulator of long-term clinical outcome in the patients is still unclear. The proteomic profile of the good- and poor outcome patients indicated a significant difference in the level of CFD during inflammation that would impact long-term outcome. The findings regarding the clinical impact of CFD and Col1a1 during inflammation mainly enhancing fibroblast migration creates a feedback loop for later healing stages. This observation is also strengthened by findings from earlier studies demonstrating that fibroblast production of collagen type I synthesis is mainly initiated during the proliferative- and remodeling healing phases (4).

Thus, the current identification of time-based opposing effects of CFD in the regulation of Col1a1 production during Achilles tendon repair may provide a possible explanation regarding the long-term effects of CFD on connective tissue repair. Unfortunately, the exact timeline of these opposing effects of CFD on Col1a1 production during the healing process cannot be determined from the current experimental construct. However, the results from the present studies do demonstrate that opposing effects are initiated during the transitional phase between inflammation, at 1 week post-injury, and proliferation at 2 weeks post-injury.

Fibroblast migration plays a vital role during tissue healing from the late inflammation stage by acting as a trigger of inflammation-proliferation transition (24, 35). Thus, the knowledge that Col1a1 after injury acts chemotactically, as a migration-related protein for fibroblasts, likely explains an essential function for CFD upregulation of Col1a1 (36). Our *in vitro* investigations established CFD as a novel regulator in this model system, which can potentially affect fibroblast migration during the transition from the inflammatory to the proliferative stage of tissue healing. Moreover, the observation that opposing concentrations of CFD during inflammation and proliferation, both associated with an up-regulated expression of Col1a1, which in turn led to improved fibroblast migration and enhanced tendon healing, seems to strengthen the concept of an important role for the molecule in the inflammation-proliferation transition.

The earlier observations at 2 weeks post-injury during the proliferative phase of healing indicated that lower CFD expression was associated with improved long-term clinical patient outcome (37), and that link has been further corroborated by the results of the present findings. The increased synthesis of Col1a1, which we observed induced by lower CFD expression during the proliferation phase, may improve ECM formation and remodeling since type I collagen is an important component of the ECM in connective tissues such as tendons and ligaments.

Migration of fibroblasts to the site of injury plays an important role during wound healing. Based on the experimental observations, we speculate that the increased Col1a1 expression associated with CFD levels during inflammation and proliferation leads to improved fibroblast migration to promote ATR repair, subsequently leading to an enhanced healing outcome.

A compromised healing process may lead to less strength of the repair tissue and limitations with regard to daily activities, which are important assessments during outcome evaluation in the clinic. An impaired fibroblast migration during the later proliferative healing phase may lead to less provisional matrix formation with Col1a1, which presumably leads to more limitations in daily life with less loading of the injured tissue, all causative factors for ATR patients having a prolonged healing time and an unsatisfactory patient-reported outcome (38). The present results indicate that an increased Col1a1 expression, presumably via fibroblast migration, improves the quality of healing by increasing the strength and stiffness in the injury area. Additionally, the long-term limitations on running, jumping, physical work activities and walking on uneven surfaces declined in association with the increased Col1a1 production during both the inflammation and proliferation phases of healing. Previous studies reported that higher and faster production of type I collagen can lead to an enhanced ATR repair (13, 16). The present observations strengthen the previous findings and provide further insights regarding Col1a1 production, cell migration and how they combine to improve ATR healing, leading to better 1-year healing outcomes.

There are several limitations for the study design, one of the main study limitation is that the proteomic profile from the inflammatory healing phase consisted of tendon tissue biopsies (i.e. were tissue-associated), while the proteomic profile from the proliferative healing phase comprised of microdialysates (i.e. were soluble molecules) from the paratenon. This may limit the comparability of proteomic profiles from the two different healing stages. However, earlier studies have demonstrated that microdialysis of the paratenon, which is a biologically active part of the tendon, can capture the proteins of the tendon tissue that control cell proliferation during tendon healing (17, 39, 40). Peritendinous microdialysates from the proliferative healing phase in patients with ATR have also captured specific tendon substances, which are predictive of patient outcome at one year (16, 17). Moreover, this study used an experimental *in vitro* construct to further characterize the opposing effects of CFD during inflammation and proliferation. In further studies it would be helpful to examine the *in vitro* results also with tenocytes, and also to add another validated inflammatory model. Another possible limitation is that multiple surgeons performed the ATR repair, which may be a possible source of bias. However, all surgeons used the same standardized operative techniques according to a predefined study protocol. A third limitation is that it is unknown whether the potential relationship between CFD/adipsin and Col1a1 involves CFD/adipsin as a zymogen or an activated protease. In addition, it is not clear what form(s) of CFD/adipsin were being detected or needed for various *in vitro* activities (zymogen, active protease, specific degradation fragments).

Taken together, the current findings further establish the potential of CFD to function as a prognostic biomarker of good human Achilles tendon healing. While currently specific to tendon healing, future studies may confirm CFD to be a generic biomarker prognostic of human dense connective tissue repair in general. The bioinformatic analysis and experimental investigation demonstrated that CFD likely promotes tissue repair by enhancing Col1a1 synthesis via in part, to increased fibroblast migration during both inflammation and proliferation. Among the 13 overlapping patients with both tissue biopsies and microdialysates, CFD is significantly higher in tendon tissues in good outcome patients while CFD microdialysate levels were significantly lower in good outcome patients. These findings follow the same pattern as observed and reported in the manuscript. Additionally, we checked the correlation between patient outcome and CFD expression among these 13 patients. We observed that CFD expression is positively associated with better patient outcome in tissues, but negatively associated with better clinical outcome in microdialysates. All these new findings matched our results which were already presented in the manuscript text, so we believe that these new findings strengthened our conclusions. But we also concur that more studies are needed. These findings may also be a highly relevant to the development of both individualized treatment protocols as well as new targeted treatments, which could enhance the long-term healing outcomes for patients suffering with dense connective tissue injuries that currently yield a poor clinical outcome.

## Data availability statement

MS proteomic data files are deposited at the ProteomeXchange Consortium (http://proteomecentral.proteomexchange.org) through the PRIDE partner repository (https://www.ebi.ac.uk/pride/) under the dataset PXD029202 and PXD033163. The extracted protein LFQ intensity and GSEA data are provided as supplementary files. Clinical data was collected from the database at the section of Orthopedics, Department of Molecular Medicine and Surgery, Karolinska Institutet.

## Ethics statement

The studies involving humans were approved by Regional Ethical Review Committee in Sweden (Reference no. 2009/2079-31/2: 2013/1791-31/3). The studies were conducted in accordance with the local legislation and institutional requirements. The participants provided their written informed consent to participate in this study.

## Author contributions

The project organization, training, resources and funding acquisition by PWA and ASA; Data analysis by JW; Data curation by JW, ZZ and JC; Visualization by JW, ZZ, DAH and ASA; Experiment completed by JC; Writing original draft by JC; Manuscript reviewing and editing by ZZ, DAH, PWA and ASA. 
