# Supplementary material for: Complement factor D regulates collagen type I expression and fibroblast migration to enhance human tendon repair and healing outcomes
Source: Front Immunol. 2023 Sep 6;14:1225957. doi: 10.3389/fimmu.2023.1225957 (PMC10512081; doi:10.3389/fimmu.2023.1225957)
Supplement: Supplementary file 1 [file DataSheet_1.docx]

Fig S1.


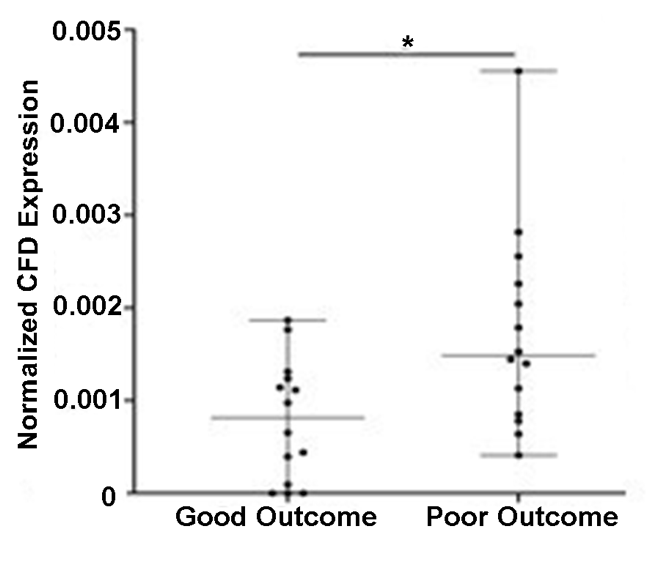


Fig S1. CFD expression among good and poor outcome patients based on proteomic profile using microdialysate.
