# Supplementary figures and images for: Complement factor D regulates collagen type I expression and fibroblast migration to enhance human tendon repair and healing outcomes
Source: Front Immunol. 2023 Sep 6;14:1225957. doi: 10.3389/fimmu.2023.1225957 (PMC10512081; doi:10.3389/fimmu.2023.1225957)

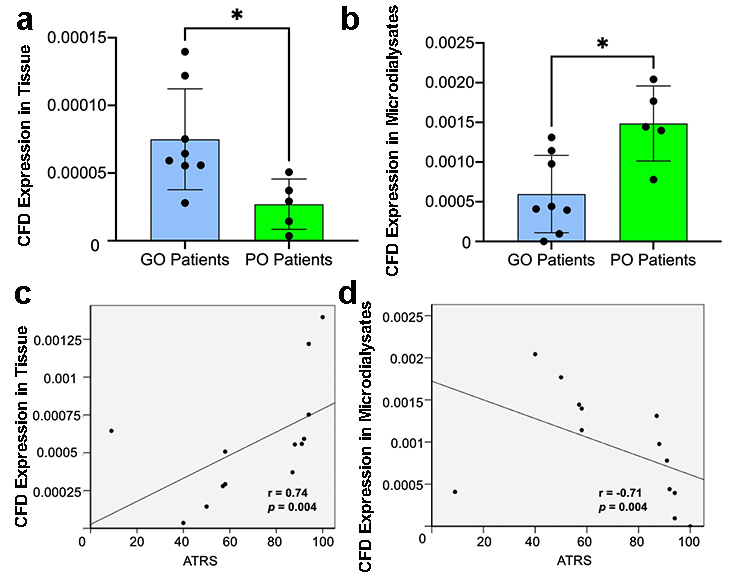

Supplement: Supplementary file 2 [file Image_1.jpeg]
